# Transcriptomic changes across the life cycle of *Trypanosoma cruzi II*

**DOI:** 10.7717/peerj.8947

**Published:** 2020-05-14

**Authors:** Lissa Cruz-Saavedra, Gustavo A. Vallejo, Felipe Guhl, Juan David Ramírez

**Affiliations:** 1 Grupo de Investigaciones Microbiológicas-UR (GIMUR), Departamento de Biología, Facultad de Ciencias Naturales, Universidad del Rosario, Bogota, Colombia; 2 Laboratorio de Investigación en Parasitología Tropical, Facultad de Ciencias, Universidad del Tolima, Ibagué, Colombia; 3 Centro de Investigaciones en Microbiología y Parasitología Tropical (CIMPAT), Facultad de Ciencias, Universidad de Los Andes, Bogota, Colombia

**Keywords:** *Trypanosoma cruzi*, Metacyclic trypomastigotes, Epimastigotes, Trypomastigotes, Transcriptomic, Pathways

## Abstract

*Trypanosoma cruzi* is a flagellated protozoan that causes Chagas disease; it presents a complex life cycle comprising four morphological stages: epimastigote (EP), metacyclic trypomastigote (MT), cell-derived trypomastigote (CDT) and amastigote (AM). Previous transcriptomic studies on three stages (EPs, CDTs and AMs) have demonstrated differences in gene expressions among them; however, to the best of our knowledge, no studies have reported on gene expressions in MTs. Therefore, the present study compared differentially expressed genes (DEGs), and signaling pathway reconstruction in EPs, MTs, AMs and CDTs. The results revealed differences in gene expressions in the stages evaluated; these differences were greater between MTs and AMs-PTs. The signaling pathway that presented the highest number of DEGs in all the stages was associated with ribosomes protein profiles, whereas the other related pathways activated were processes related to energy metabolism from glucose, amino acid metabolism, or RNA regulation. However, the role of autophagy in the entire life cycle of *T. cruzi* and the presence of processes such as meiosis and homologous recombination in MTs (where the expressions of SPO11 and Rad51 plays a role) are crucial. These findings represent an important step towards the full understanding of the molecular basis during the life cycle of *T. cruzi*.

## Introduction

*Trypanosoma cruzi* is a flagellated protozoan that causes Chagas disease, and it is estimated to affect approximately 8 million individuals worldwide (OMS, 2019; Rassi & De Rezende, 2012). *T. cruzi* has a complex life cycle comprising four well-differentiated morphological stages—epimastigote (EP), amastigote (AM), cell-derived trypomastigote (CDT) and metacyclic trypomastigote (MT), that circulate among several mammals, such as humans and vectors from the Reduviidae family (Goldenberg & Avila, 2011).

The life cycle of *T. cruzi* in vectors initiates when an insect consumes it from a mammal that possesses the CDT stage in the peripheral blood. CDTs are characterized by their mobility and inability to replicate. They migrate through the insect gut until they reach the midgut where they replicate and differentiate into mobile EPs. Subsequently, EPs move through the intestine while undergoing multiple rounds of replication via binary fission. Finally, in the rectal blister of the insect and because of nutritional stress, EPs adhere to the perimicrovilar film of the epithelial cells of the insect and transform into MTs, which are the infectious stages of *T. cruzi*. They are characterized by their mobility but not replication. MTs are dejected with the feces of the insect to infect other mammals (Goldenberg & Avila, 2011; Teixeira et al., 2012). The process through which EPs transform into MTs is called metacyclogenesis, and it involves multiple changes in the parasite such as modifications in nucleus and the kinetoplast location, increases in heterochromatin, elongation of the cytoplasm, increases in the flagellum pocket in the cytoplasm, and increases in the expressions of proteins mainly associated with virulence (e.g., transialidase-like GP63, mucins and mucin-associated surface proteins (MASP)) (Avila et al., 2003; Bayer-Santos et al., 2013; Contreras, Morel & Goldenberg, 1985; Ferreira et al., 2008).

On the other hand, the life cycle of *T. cruzi* in mammals initiates when MTs that are dejected in the feces of the insect reach the bloodstream of mammals either through laceration generated by the bite of the insect or via other routes such as oral, transfusion, congenital, or laboratory accidents, and they subsequently infect mononucleated or epithelial cells near the infection site. Once in the bloodstream, the parasites inside the white cells differentiate into nonmobile replicative forms called AMs via a process called amastigogenesis. Similar to EPs, AMs undergo multiple rounds of replication and finally they differentiate into nonreplicative mobile forms—CDTs, which lyse the cell and migrate through the bloodstream to invade other mononucleated cells and/or tissues for which they have a high tropism (e.g., the heart, esophagus, colon and adipose tissue as well as the nervous tissue in some cases). The progression of infection in these tissues is directly responsible for the clinical manifestations of Chagas disease (Cruz et al., 2015; Cucunubá et al., 2016; Ferreira et al., 2011; Goldenberg & Avila, 2011; Hernández et al., 2014).

Recently, the importance of autophagy in controlling the life cycle of *T. cruzi* has been described (Salassa & Romano, 2019). Studies have indicated that the activation of this metacyclogenesis process is triggered by autophagy, and although its participation has been inferred during the entire life cycle of *T. cruzi*, the regulatory mechanism of this process in all the stages of *T. cruzi* is unknown to date (Salassa & Romano, 2019; Vanrell et al., 2017). However, one of the great uncertainties during the life cycle of this parasite is associated with gene regulation, particularly considering polycistronic transcription and the absence of transcription initiation sites where most of the regulatory processes occur at the post-transcriptional level. Some studies have demonstrated the influence in the expression profiles of ribosomal proteins between MTs and EPs, nevertheless, similar to the role of autophagy, to date, the changes in the life cycle of this parasite have not been completely elucidated (Smircich et al., 2015). These are only a few processes that are believed to play a relevant role in the life cycle of *T. cruzi*; however, previous studies have demonstrated transcriptomic level differences using next-generation sequencing in EPs, CDTs and AMs. However, transcriptomic level differences in CDTs and MTs, which are two stages with similar morphological characteristics (in which some stages of the *T. cruzi* life cycle are considered to perform the same function), remain unknown. Moreover, to the best of our knowledge, studies comparing EPs and MTs and investigating how metacyclogenesis influences the infective properties and metabolism in MTs have not been conducted to date (Belew et al., 2017; Berná et al., 2017; Dos Santos et al., 2018; Houston-Ludlam, Belew & El-Sayed, 2016; Li et al., 2016).

Therefore, there is a current need to comprehend gene regulation in the life cycle of *T. cruzi* and transcriptional profiles at different stages of this parasite to understand the pathogenicity and ability of this parasite to adapt to different microenvironments. Therefore, the objectives of the present study were to evaluate differentially expressed genes (DEGs) in MTs, CDTs, AMs and EPs in *T. cruzi* II, mainly focusing on processes such as autophagy, ribosomal profiling, and other processes (such as meiosis and homologous recombination (HR)) that have not yet been fully described in *T. cruzi*.

## Methods

### Parasite cultures

Epimastigote cultures of the strain MHOM/BR/53/Y were maintained in a liver infusion tryptose medium (LIT) supplemented with 10% fetal bovine serum. When an abundant logarithmic phase culture was obtained, a protocol standardized by our group was followed (Cruz-Saavedra et al., 2017). Briefly, an EP culture of 1 × 10^7^ cells was prepared and incubated at 26 °C. Six days post-culture, the time needed for the appearance of MT due to nutritional stress and the purification of MTs via ion-exchange chromatography of sepharose-DEAE were investigated according to the protocol of Cruz-Saavedra et al. (2017). MTs obtained in the eluate were washed twice with 1X phosphate-buffered saline (PBS), and their morphological stages were verified by observation under an optical microscope.

### RNA extraction and RNA sequencing

RNA was extracted from purified MTs using the RNeasy Plus Mini Kit (Qiagen, Hilden, Germany) according to the manufacturer’s protocol. The concentration and quality of the obtained RNA were evaluated by measurements using a nanodrop. A concentration greater than 1 mg/mL and a 260/280 index of 2 ± 2 were acceptable. In addition, the integrity of the obtained RNA was verified by electrophoresis on an agarose gel. RNAs that met these parameters were sent for sequencing to Novogene Bioinformatics Technology Co., Ltd., Beijing, China, using the Illumina HiSeq X-TEN platform, Novogene performed a verification of the RNA quality on Qubit Assays and electrophoresis on agarose gels. Strand-specific TrueSeq RNAseq Library Prep with an insert size of 350 bp was selected to prepare RNA libraries, and the size of each read was 2 × 150 bp. In addition, in order to guarantee the quality of the reads that will be used in the analysis, the reads containing adapters, reads containing *N* > 10% (N represents the base cannot be determined) and reads containing low quality (*Q*score <= 5) base which is over 50% of the total base were all removed. This procedure is part of the technical service performed by Novogene Bioinformatics Technology. From the filtered file, according to the parameters mentioned previously, the quality of these reads was determined using the fastQC software (http://www.bioinformatics.babraham.ac.uk/projects/fastqc/). In summary, parameters such as per base quality score, per base sequence, GC content and Kmer content were evaluated (Table S1).

### Obtaining the reads of EPs and CDTs

Twelve paired-end reads for the EPs, CDTs and AMs of the strain MHOM/BR/53/Y resulting from sequencing on the Illumina HiSeq 1000 platform were available and obtained from the European Nucleotide Archive (ENA) under the PRJNA251583 and PRJNA251582 projects. PRJNA251583 was submitted to the ENA database on 1 June 2015, by Host-Pathogen Genomics Laboratory, University of Maryland (HPGL-UMD), and the results obtained were published in the article entitled “Comparative Transcriptome Profiling of Human Foreskin Fibroblasts Infected with the Sylvio and Y Strains of *T. cruzi* ” (Houston-Ludlam, Belew & El-Sayed, 2016). On the other hand, PRJNA251583 was submitted to the ENA database on 1 January 2015, by Host-Pathogen Genomics Laboratory-University of Maryland (HPGL-UMD), and as a result the paper “Transcriptome Remodeling in *T. cruzi* and Human Cells during Intracellular Infection” (Li et al., 2016). The information about the files used is available in Table S1. After downloading these files, the reads were verified using the fastQC software and the abovementioned parameters (Table S1).

### Differential expression mapping and analysis

Twelve paired-end reads files obtained for MTs, EPs, AMs and CDTs (three biological replicates per stage), were aligned using the read alignment program TopHat version v2.1.0 under default parameters. TopHat uses as an alignment engine “bowtie” but also has the ability to review unallocated reads and align them using the information on splicing junctions. To perform the analysis Tophat was provided of the reference genome of SylvioX10-1 of *T. cruzi* in the TriTrypDB-46_TcruziSylvioX10-1 version available in EupathDB. The reference genome was obtained and indexed using the bowtie2-build option, and it was used to perform the assembly, only mapped reads were considered for subsequent analysis. Files obtained from the TopHat output were used for mapping in Cufflink (Trapnell, Pachter & Salzberg, 2009), whereas those obtained from the previously described assembly were used for the gene coordinates in the gff file TriTrypDB-46_TcruziSylvioX10-1 available in EupathDB to obtain the gene coordinates in the reference genome and matched against the reference gff annotation using the cufflinks tool.

Subsequently, a comparison between the biological replicates for each stage and between the organisms in each stage (MT, EP and CDT) was performed using the cuffmerge tool. Finally, fragments per kilobase of exon per million fragments mapped (FPKM) was used to normalize the RNA expression. The fold changed was calculated for each comparison, considering a *q*-value of <0.05 for determining shortfall deviation risks, differential gene expressions were calculated using the cuffdiff tool under the number of threads (p), labels (L) and multi-read-correct (u) options, finally, the FDR-adjusted *p*-value (*q*-value) correction was performed (Trapnell, Pachter & Salzberg, 2009; Trapnell et al., 2012). Additionally, all data were plotted using the CummeRbund software package R, including Volcano graph, Multi-Dimensional Scaling (MDS) plot, heat maps, and Shannon divergence or Jensen–Shannon distance (JS distance) analysis (Trapnell et al., 2012).

### Gene ontology and pathway reconstruction

From the tables obtained when performing differential expression analysis in cuffdiff, which also contained the information of the down-regulated and up-regulated DEGs IDs when comparing gene expression among MTs and AMs, MTs and CDTs and MTs and EPs (Table S2, gene column). We constructed lists of DEGs IDs and these were submitted to the EupathDB TriTryp online tool in six different files; two per stage comparison (down-up-regulated). Two files were obtained for each comparison and type of expression (down-up regulated) from the analysis in the EupathDB TriTryp tool. The first one contained the list with the gene ontology terms for the DEGs that were subsequently deposited in Table S3, these were obtained using the download-function prediction-GO terms tool, and a second, corresponding to a FASTA file containing the coding sequence for each of the DEGs (*q* value < 0.05) that was used as input for the Kyoto Encyclopedia of Genes and Genomes (KEGG) Automatic Annotation Server (KAAS) tool, for that purpose the tool used was download-FASTA. Six FASTA files in total were obtained, these files were used as input files for reconstructing signaling pathways in KAAS (Aurrecoechea et al., 2017; Warrenfeltz et al., 2018). Each gene FASTA file (six) was submitted to the KAAS tool, the GHOSTX homology search algorithm and the GENES dataset for kinetoplastids available in the manually curated KEGG GENES database. KAAS is a manual annotator that allows using the information from the FASTA files to determine the genes that encode these sequences by means of alignment against the databases available in KEGG that contain the information of trypanosomatids genes, and subsequently, perform a reconstruction of the signaling pathways, in which these genes are participating. Based on this strategy, we performed the reconstruction of signaling pathways from DEGs (Aurrecoechea et al., 2017; Moriya et al., 2007). The html file obtained was analyzed under the “Pathway” map option to search the pathways that presented a greater KEGG. This tool allowed predicting the mapping for each differentially regulated pathway and grouped them according to the signaling pathways in which they are activated, thus allowing their graphical visualization. The Adobe Photoshop CC software was used to condense all the data obtained into a single graph, which included down-regulated and up-regulated genes in the three biological stages (Moriya et al., 2007). In addition, Excel lists were generated for all the data produced by KAAS to compare the down-regulated pathways between MTs and AMs, MTs and CDTs and between MTs and EPs as well as the number of differentially regulated genes. Subsequently, the data were plotted for 15 pathways that presented the highest number of down-regulated and up-regulated DEGs in each comparison.

## Results

### Differential gene expression during the life cycle of *T. cruzi*

The raw sequence reads, for the 12 transcriptomes included in this study, had an average of 41442480,75 bases and a standard deviation of 16660041,89 bases. The results for each of the treatments and replicates are available in Table S1. No differences were detected between the replicates of AMs, CDTs, EPs and MTs (Fig. S1).

**Figure 1:**
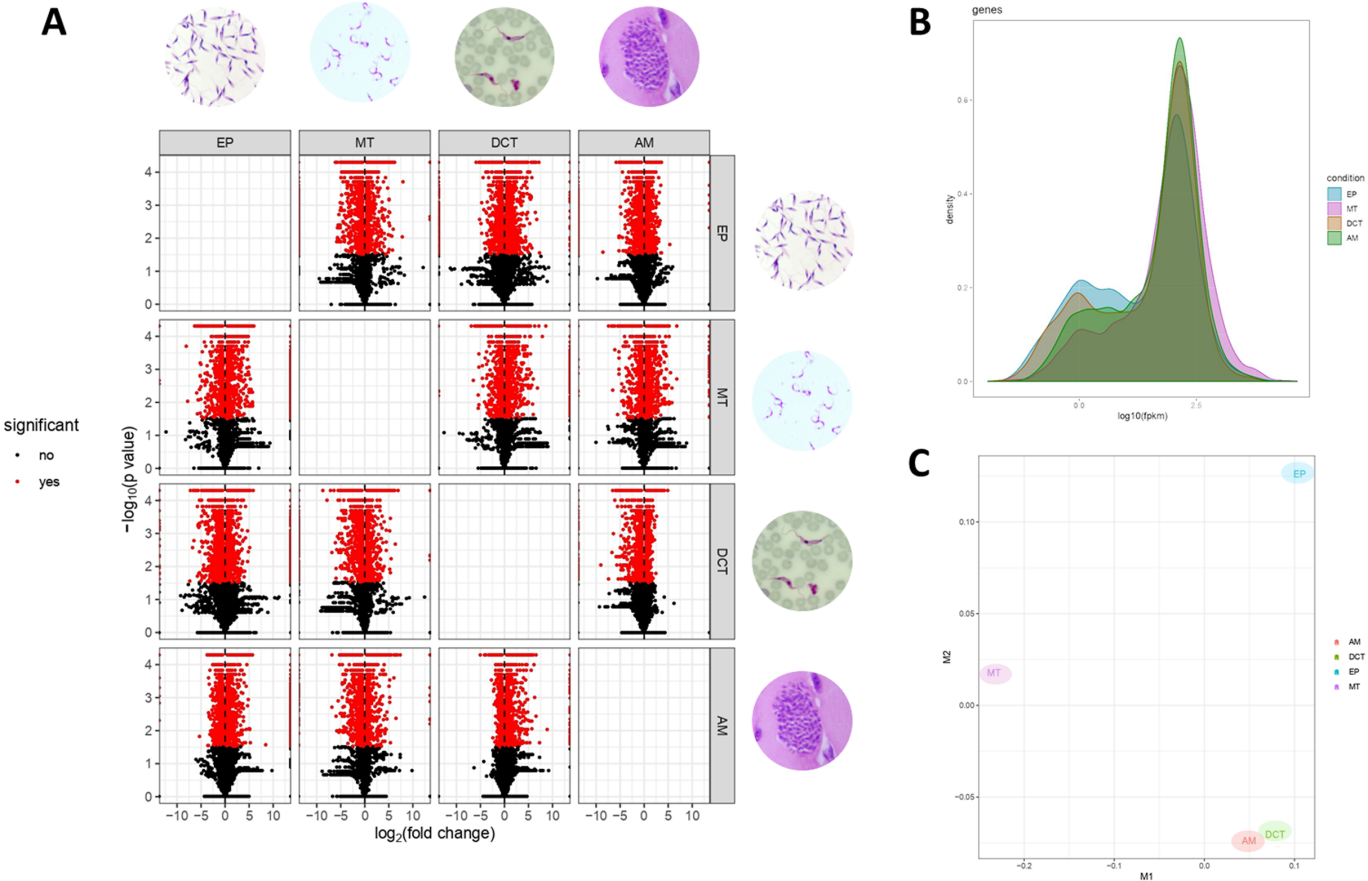
Gene expression in epimastigotes, CDTs, amastigotes and metacyclic trypomastigotes. (A) Volcano plot comparing differentially expressed genes (DEGs) among epimastigotes, amastigotes, cell-derived trypomastigotes, and metacyclic trypomastigotes. (B) The distribution analysis of FPKM values across epimastigotes, amastigotes, cell-derived trypomastigotes, and metacyclic trypomastigotes. (C) Multi-Dimensional Scaling plot (MDS) across epimastigotes, amastigotes, cell-derived trypomastigotes, and metacyclic trypomastigotes.

**Figure 2:**
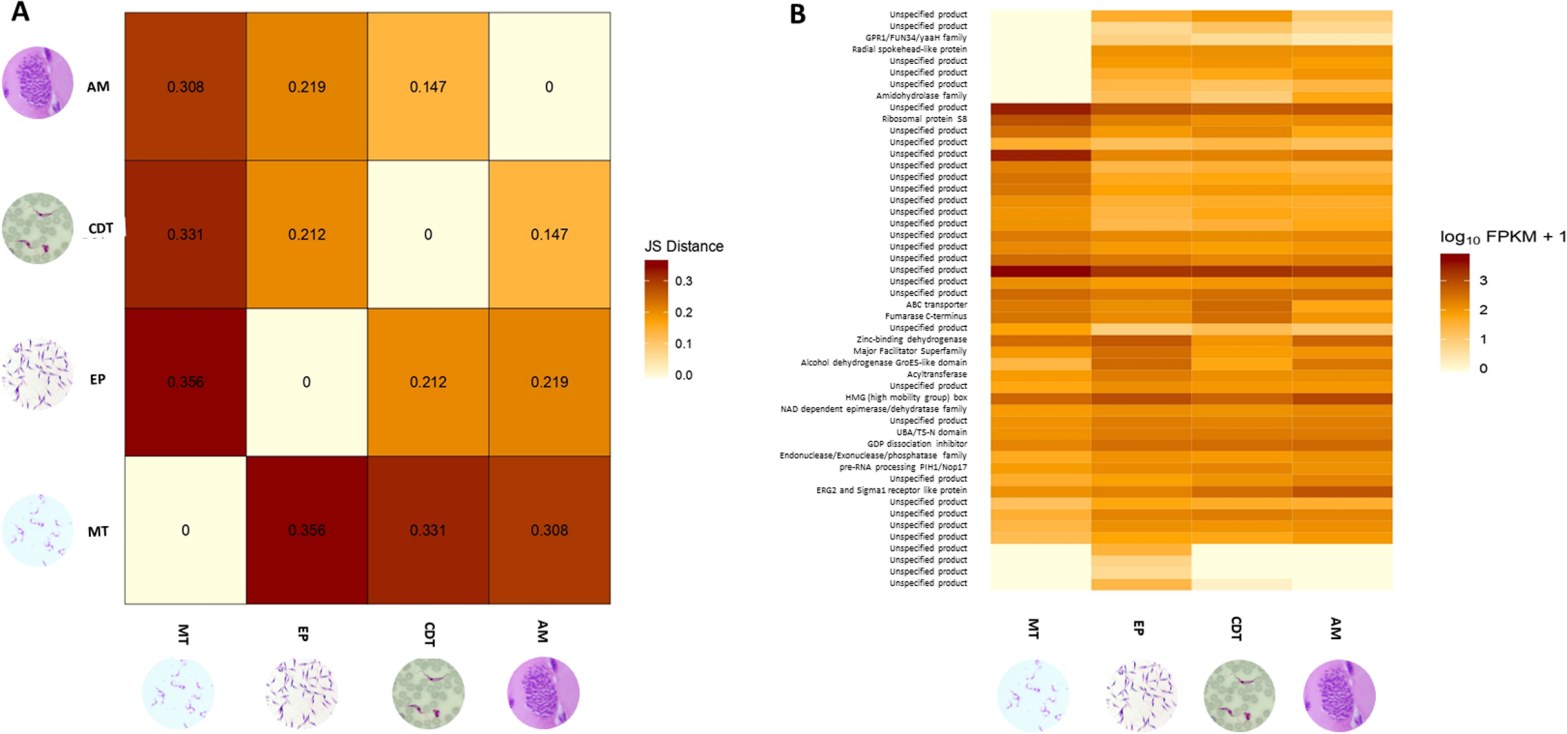
Analysis of differentially expressed genes (DEGs). (A) Jensen–Shannon distance (JS distance) analysis among epimastigotes, amastigotes, cell-derived trypomastigotes, and metacyclic trypomastigotes. (B) The 50 most up-regulated DEGs among epimastigotes, amastigotes, cell-derived trypomastigotes and metacyclic trypomastigotes.

Differences in gene expressions were observed among the three stages of *T. cruzi* (Fig. 1A). When CDTs were compared with MTs, 2,108 genes were down-regulated and 1,972 were up-regulated, whereas when EPs were compared with MTs, 1,739 were down-regulated and 2,188 were up-regulated, and AMs compared to MTs, 1,732 were down-regulated and 1,917 were up-regulated (Table S2). The distribution analysis of FPKM values across individual samples revealed that transcriptomes with the highest number of genes and the highest FPKM value corresponded to MTs, followed by AMs and CDTs, whereas the lowest FPKM value was observed for EPs and MTs (Fig. 1B). The Multi-Dimensional Scaling (MDS) plot shows a greater dissimilarity between MTs, EPs, maintaining this when comparing the transcriptome of the previous stages with AMs and CDTs, on the contrary, of AMs and CDTs have a high similarity between them (Fig. 1C). Interestingly, the Jensen–Shannon divergence or JS distance analysis revealed that the lowest differentiation value was 0.147 between AMs and CDT, followed by 0.212 between EPs and CDTs, 0.308 between AMs and MTs, 0.331 between CDTs and MTs, and finally, 0.353 between EPs and CDTs, indicating that the similarity between MTs and CDTs is not as high as that suggested previously (Fig. 2A).

An analysis of the 50 most down and up-regulated DEGs among the three stages of *T. cruzi* revealed that 33 of these genes encoded for unspecified products, and the remaining 17 genes for different functions, including the ABC transporter, preRNA processing PIH1/Nop17, ribosomal protein S8, and GPR1/FUN34/yaaH family (Fig. 2B). Therefore, to determine if these genes are associated with any specific signaling pathway, we reconstructed the signaling pathways in KAAS. However, we found only two genes associated with ribosomal coding for the small subunit ribosomal protein S15Ae (TcSYL_0001610) and the large subunit ribosomal protein LP1 (TcSYL_0000480), whereas the other signaling pathways had only one DEG. Of note, one of these pathways was associated with HR and the coding gene for topoisomerase (DNA) II binding protein 1 (TcSYL_0001820), which is specific to this process.

### Differentially regulated signaling pathways

**Figure 3:**
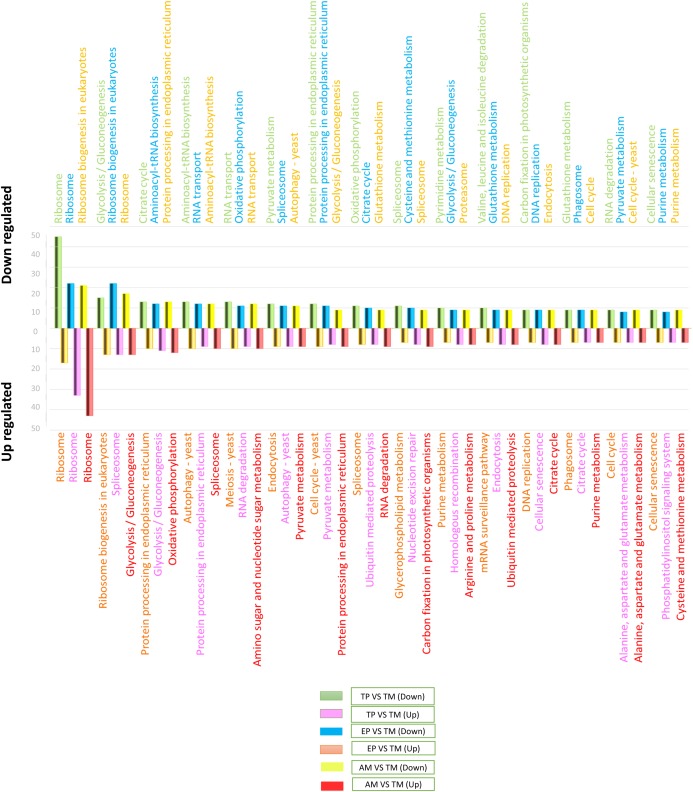
The 15 most differentially regulated pathways. Comparison of the down-regulated (green) and up-regulated (pink) pathways between metacyclic trypomastigotes and cell-derived trypomastigotes. Comparison of the down-regulated (blue) and up-regulated (orange) pathways between metacyclic trypomastigotes and epimastigotes. Comparison of the down-regulated (yellow) and up-regulated (red) pathways between metacyclic trypomastigotes and amastigotes.

To determine the signaling pathways associated with DEGs, the results obtained in KAAS were used, which included the signaling pathways and the number of DEGs. Data analysis was performed using Excel and six comparisons were evaluated: down-regulated genes in AMs vs. CDTs, up-regulated genes in AMs vs. CDTs, down-regulated genes in MTs vs. CDTs, up-regulated genes in MTs vs. CDTs, down-regulated genes in MTs vs. EPs, and up-regulated genes in EPs vs. MTs. The data corresponding to the gene ontology is shown in Table S3. In all the groups, the pathway with the highest number of DEGs was associated with ribosomes. A total of 45 genes in this pathway were down-regulated and 17 were up-regulated in MTs compared with those in CDTs. In contrast, when gene expression was compared between MTs and EPs, the number of up-regulated DEGs was greater (*n* = 33) than the number of down-regulated genes (*n* = 22), and 17 genes were down-regulated and 43 up-regulated between MTs and AMs (Fig. 3). Furthermore, the signaling pathway for ribosome biogenesis in eukaryotes was up-regulated only in MTs compared with that in CDTs and AMs (*n* = 13, 21) and it was down-regulated in MTs compared with that in EPs (*n* = 22) (Fig. 3).

The signaling pathway for endocytosis presented seven down-regulated and nine upregulated genes when MTs were compared with CDTs, nine downregulated between MTs and AMs, and eight down-regulated and up-regulated genes when MTs were compared with EPs. The production of phagosomes exhibited similar expression patterns with seven down-regulated and up-regulated genes when MTs were compared with CDTs and nine down-regulated and five up-regulated genes when MTs were compared with EPs. However, these results were not found when comparing between MTs and AMs.

On the other hand, in comparison with the signaling pathways associated with energy metabolism, the signaling pathway for glycolysis exhibited 15 down-regulated and four up-regulated genes in MTs compared with that in CDTs, nine down-regulated and 12 up-regulated genes in MTs compared with that in AMs and 10 down-regulated and 11 up-regulated genes in MTs compared with that in EPs. The signaling pathway for the pyruvate metabolism, which produces pyruvate, was also differentially regulated. Overall, 12 genes were down-regulated and four were up-regulated between MTs and CDTs, whereas nine genes were down-regulated and eight were up-regulated between MTs and EPs, and nine up-regulated genes between MTs and AMs. Acetyl-CoA is produced from pyruvate, and this molecule is necessary for citric acid or Krebs cycle. As expected, the signaling pathway for Krebs cycle was also differentially regulated; the number of down-regulated and up-regulated genes was 13 and five, respectively, in MTs compared with CDTs, whereas the number of down-regulated and up-regulated genes was 11 and seven, respectively, in MTs compared with EPs, and seven genes were up-regulated when comparing MTs and AMs. Finally, from the NADH molecules produced in Krebs cycle, the available H+ is used to produce ATP during oxidative phosphorylation, which is the main source of energy in most cells. We observed 11 down-regulated genes and four up-regulated genes that were differentially expressed in this pathway in MTs vs. CDTs, 12 down-regulated and six up-regulated genes in MTs vs. EPs, and 10 up-regulated genes in MTs vs. AMs (Fig. 3). The metabolisms of purines and pyrimidines were also differentially regulated; 10 genes were down-regulated and two were up-regulated in MTs compared with those in CDTs for pyrimidine metabolism. Conversely, when comparing this same stage (MT) with EP, the number of up-regulated genes was greater (*n* = 6) than the number of down-regulated genes (*n* = 3), however, the same results were not observed for the comparison between MTs and AMs. Similarly, the number of genes associated with purine metabolism was quite similar for all the stages, wherein the gene numbers ranged from 6 to 8, unlike the comparison between MTs and AMs where only 7 up-regulated genes were observed (Fig. 3).

One of the most important processes in the life cycle of *T. cruzi* is related to the posttranscriptional regulation of this parasite, particularly the transcriptional characteristics of the down-regulated and up-regulated genes in *T. cruzi*. In the RNA-associated pathways, 13 genes were down-regulated and 12 were up-regulated between MTs and CDTs and six were down-regulated, five were up-regulated between MTs and EPs and 12 were up-regulated to MTs and AMs. In contrast, the signaling pathway responsible for RNA degradation exhibited 9 down-regulated and three upregulated genes between MTs and CDTs. However, the opposite was observed between MTs and EPs, where the number of overregulated genes (*n* = 9) was greater than the number of down-regulated genes (*n* = 5), similar results were observed for MTs and AMs where nine up-regulated genes were found. The mRNA surveillance pathway was mainly up-regulated (*n* = 7 genes) in MTs compared with that in CDTs. However, three down-regulated genes were also detected, and the numbers were much closer to those observed in the downregulation, with eight down-regulated and four up-regulated genes (Fig. 3).

Several signaling pathways related to amino acids metabolisms were differentially regulated. One of the signaling pathways with the highest number of DEGs was the aminoacyl-tRNA biosynthesis pathway, where 13 and 15 genes were down-regulated between MTs and CDTs and between MTs and EPs, respectively, and six and five genes were up-regulated, respectively, in contrast, just 12 genes were down-regulated to MTs and AMs. In the valine signaling pathway degrading leucine and isoleucine, 10 genes were down-regulated and two were up-regulated between MTs and CDTs. In the comparison between MTs and EPs, a different expression response was observed where four genes were down-regulated and four were up-regulated. Similarly, the amino sugar and nucleotide sugar metabolism pathways were down-regulated in MTs compared with those in CDTs, with a total of eight down-regulated genes and two up-regulated between them. In contrast, a comparison between MTs and EPs showed six up-regulated and three down-regulated genes in MTs and a comparison between MTs and AMs nine up-regulated genes. Glutathione comprises three amino acids: glutamate, cysteine and glycine. The down-regulation of nine genes and the overregulation of six genes related to their metabolism were observed between MTs and CDTs. When MTs were compared with EPs, similar results were observed where nine genes were down-regulated and five were up-regulated and when we compared MTs and AMs nine genes were down-regulated, suggesting the importance of this molecule in the life cycle of *T. cruzi* (Fig. 3).

Finally, one of the altered processes that did not show differential expression was protein processing in the endoplasmic reticulum. A total of 12 genes were down-regulated and 10 genes were up-regulated between CDTs and MTs, whereas 11 were down-regulated and nine were up-regulated between MTs and CDTs and 13 up-regulated genes between MTs and AMs, indicating the importance of this pathway throughout the life cycle of the parasite. Another route with similar expression characteristics was related to spliceosomes, where 11 and eight genes were down-regulated and up-regulated between MTs and CDTs, respectively, and 11 and 13 were down-regulated and up-regulated between MTs and EPs and finally, nine were down-regulated and 10 up-regulated between MTs and AMs (Fig. 3).

### Reconstruction of signaling pathways

Based on the number of DEGs present in the differentially regulated signaling pathways and the importance of these factors in the life cycle of *T. cruzi*, we decided to reconstruct the signaling pathways and the genes present in them that were differentially regulated in MTs compared with those in CDTs and EPs.

### Autophagy

**Figure 4:**
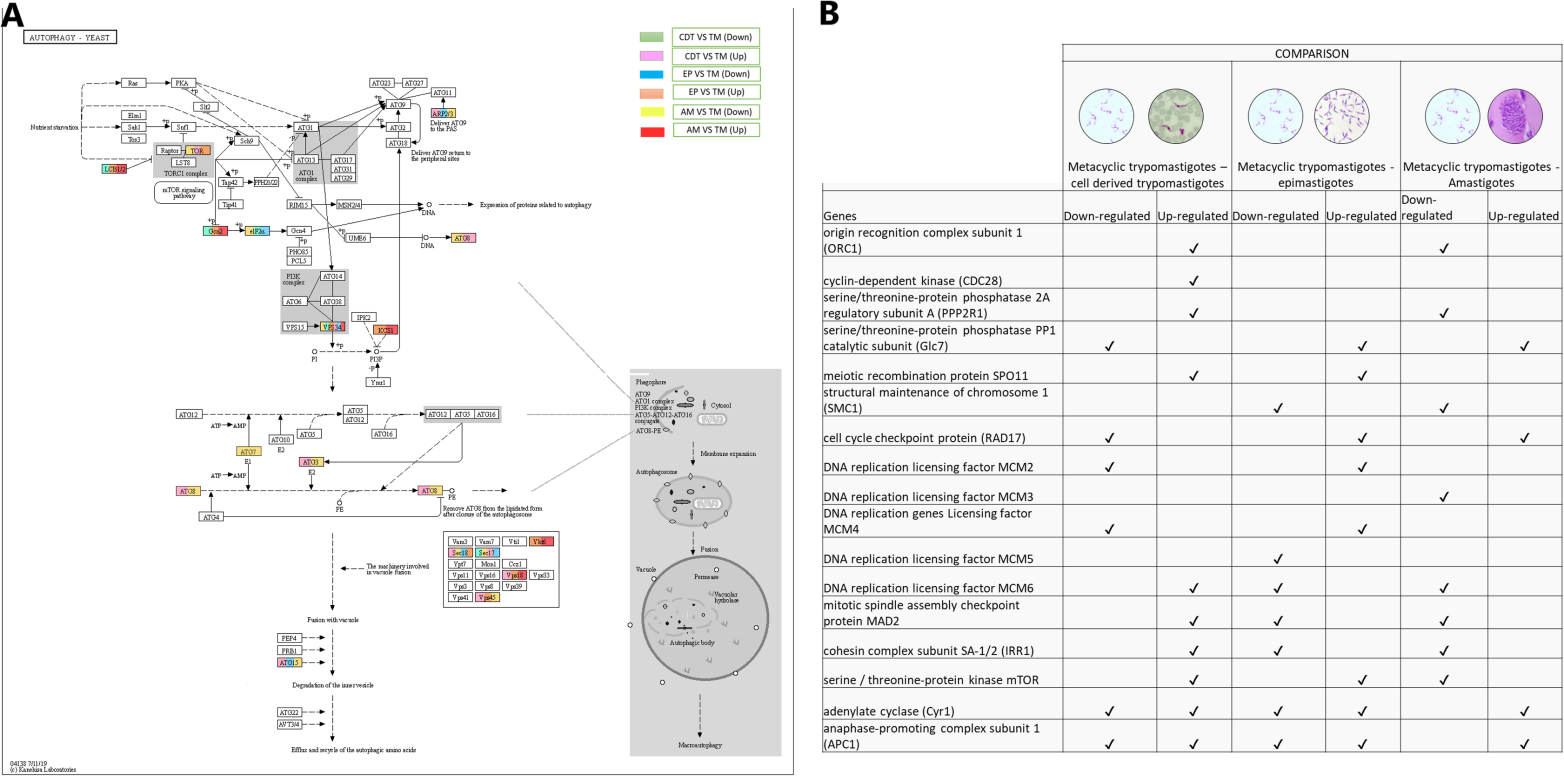
Autophagy processes. (A) Comparison of the down-regulated (green) and up-regulated (pink) pathways between metaciclic trypomastigotes and cell derived trypomastigotes. Comparison of the down-regulated (blue) and up-regulated (orange) pathways between metacyclic trypomastigotes and epimastigotes. Comparison of the down-regulated (yellow) and up-regulated (red) pathways between metacyclic trypomastigotes and amastigotes. (B) Table of down- and up-regulated genes between metacyclic trypomastigotes and amastigotes, cell derived trypomastigotes, epimastigotes. The pathway annotation pipeline using KAAS for KEGG pathway assignments was used to reconstruct the pathways and was modified to include down- and up-regulated genes (Kanehisa & Goto, 2000).

Autophagy has recently been associated with the control and differentiation of the life cycle of *T. cruzi*. Therefore, we decided to evaluate the changes in gene expressions that occurred among the life cycle stages. The regulation process with the highest number of up-regulated genes was observed when MTs were compared with CDTs and MTs compared with AMs. These genes included GABA (A) receptor-associated protein (ATG8- TcSYL_0079440) and ubiquitin-like-conjugating enzyme ATG3 (TcSYL_0114940). They were up-regulated between MTs and EPs, and MTs and AMs for the gene encoding inositol-hexakisphosphate 5-kinase (KCS1-EC: 2.7.4.21). On the other hand, two genes were down-regulated between MTs and CDTs and up-regulated between MTs and EPs and MTs and AMs. These were serine palmitoyltransferase (LCB1/2-EC: 2.3.1.50-TcSYL_0132770) and eukaryotic translation initiation factor 2-alpha kinase 4 (Gcn2-EC: 2.7.11.1-TcSYL_0075610). The translation initiation factor 2 subunit 1 was down-regulated in MTs compared with that in CDTs, EPs, AMs. The serine/threonine-protein kinase mTOR (TOR-EC: 2.7.11.1-TcSYL_0047210) and vacuolar protein sorting-associated protein 45 (VSP45-TcSYL_0011600) were up-regulated in MTs compared with those in CDTs and EPs and down-regulated in MTs between AMs. Lipase ATG15 (ATG15-EC: 3.1.1.3) and actin-related protein 2 (ARP2/3) were up-regulated in MTs compared with that in CDTs, whereas they were down-regulated in MTs compared with those in EPs and MTs compared with AMs. The genes coding for vesicle-fusing ATPase (SEC18 - TcSYL_0110800) and alpha-soluble NSF attachment protein (SEC17 - TcSYL_0118420) showed different expression profiles. The former was up-regulated between MTs and CDTs between MTs and EPs and down-regulated between MTs and AMs and between MTs and EPs; the latter was down-regulated between MTs and CDTs, between MTs and AMs and between MTs and EPs and they were up-regulated between MTs and EPs. The Lipase ATG15 (TcSYL_0196430) were down-regulated between MTs and EPs, and between MTs and AMs and up-regulated in MTs and CDT. Finally, the phosphatidylinositol 3-kinase gene (EC: 2.7.1.137) was differentially regulated in all the comparisons (VSP34 - TcSYL_0103210) (Fig 4).

### Ribosomal profiles

**Figure 5:**
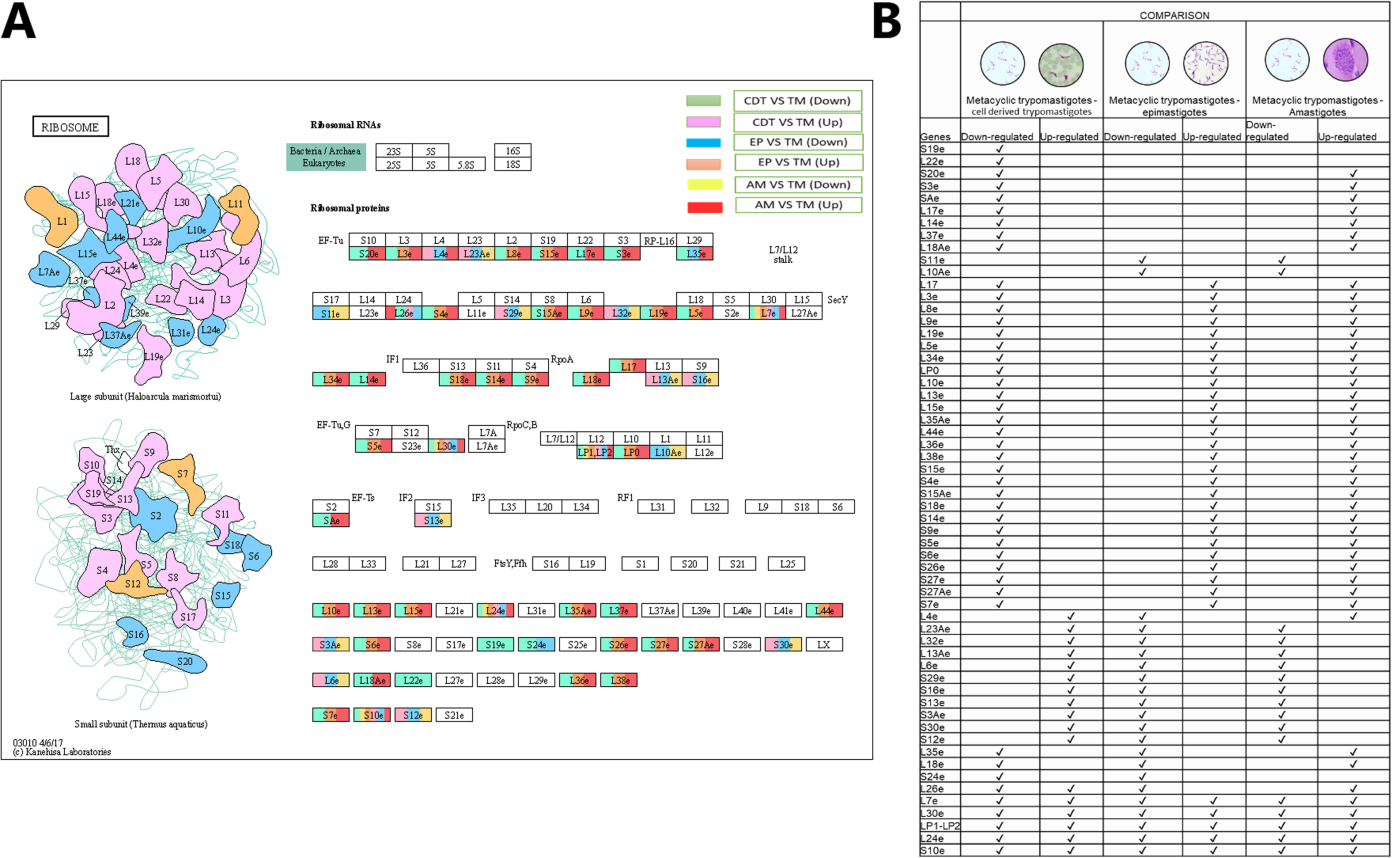
Ribosomal profiles. (A) Comparison of the down-regulated (green) and up-regulated (pink) pathways between metacyclic trypomastigotes and cell derived trypomastigotes. Comparison of the down-regulated (blue) and up-regulated (orange) pathways between metacyclic trypomastigotes and epimastigotes. Comparison of the down-regulated (yellow) and up-regulated (red) pathways between metacyclic trypomastigotes and amastigotes. (B) Table of down- and up-regulated genes between metaciclic trypomastigotes and amastigotes, cell derived trypomastigotes, epimastigotes. The pathway annotation pipeline using KAAS for KEGG pathway assignments was used to reconstruct the pathways and was modified to include down-regulated and up-regulated genes (Kanehisa & Goto, 2000).

As mentioned previously, one of the signaling pathways that presented the greatest number of down-regulated and up-regulated DEGs in the three life cycle stages of *T. cruzi* was associated with ribosomes. Previous studies have demonstrated the importance of ribosomal profiles as a possible mechanism of posttranscriptional regulation. To determine the specific genes with differential expressions and if they were shared between stages and the types of expression (up or down), the data obtained by KAAS shown in Fig. 4 was condensed. The results showed that small subunit ribosomal proteins S20e (TcSYL_0042980), S3e (TcSYL_0010030), SAe (TcSYL_0121230), and large subunit ribosomal proteins L17e (TcSYL_0048680), L14e (TcSYL_0200830), and L37e (TcSYL_0138350) were down-regulated only in MTs compared with those in CDTs and up-regulated in MTs compared with AMs. S19e (TcSYL_0045890) and L22e (TcSYL_0005370) were only down-regulated in MTs compared with those in CDTs. The small subunit ribosomal protein S11e (TcSYL_0118400) and large subunit ribosomal protein L10Ae (TcSYL_0014830) were down-regulated in MTs compared with those in EPs and AMs compared with MTs. No other differentially regulated gene showed this characteristic of exclusivity between expression classes in the stages of *T. cruzi*. However, some genes were down-regulated in MTs compared with those in CDTs and up-regulated in MTs compared with those in EPs and AMs. These genes included large subunit ribosomal proteins L3e (TcSYL_0045960), L8e (TcSYL_0203300), L9e (TcSYL_0140530), L19e (TcSYL_0078610), L5e (TcSYL_0115310), L34e (TcSYL_0015800), LP0 (TcSYL_0083010), L10e (TcSYL_0147240), L13e (TcSYL_0001000), L15e (TcSYL_0140770), L35Ae (TcSYL_0045350), L44e (TcSYL_0088040), L36e (TcSYL_0074510), and L38e (TcSYL_0001390) and small subunit ribosomal proteins S15e (TcSYL_0201640), S4e (TcSYL_0089620), S15Ae (TcSYL_0001610), S18e (TcSYL_0113930), S14e (TcSYL_0140720), S9e (TcSYL_0114280), S5e (TcSYL_0074870), S6e (TcSYL_0115180), S26e (TcSYL_0043730), S27e (TcSYL_0014810), and S27Ae (TcSYL_0113470). In contrast, a group of genes up-regulated in MTs compared with those in CDTs and down-regulated in MTs compared with those in EPs and AMs was also observed among the large subunit ribosomal proteins L4e (TcSYL_0003330), L23Ae (TcSYL_0078780), L32e (TcSYL_0115140), L13Ae (TcSYL_0050090), and L6e (TcSYL_0009100) and the small subunit ribosomal proteins S29e (TcSYL_0180240), S16e (TcSYL_0023130), S13e (TcSYL_0087830), S3Ae (TcSYL_0132400), S30e (TcSYL_0168900), and S12e (TcSYL_0108040), except L4e (TcSYL_0003330) that was up-regulated in MTs between AMs. Large subunit ribosomal proteins L35e (TcSYL_0156520) and L18e (TcSYL_0005640) and small subunit ribosomal protein S24e (TcSYL_0073760) were down-regulated in both MTs vs. CDTs and MTs vs. EPs and up-regulated in MTs vs. AMs. The coding gene for large subunit ribosomal protein L26e (TcSYL_0103320) was not up-regulated in MTs compared with that in EPs, but was differentially regulated for the other comparisons and up-regulated to MTs between AMs. Finally, some genes were present in all regulation processes and in all stages. These included the large subunit ribosomal proteins L7e (TcSYL_0022670), L30e (TcSYL_0132850), LP1-LP2 (TcSYL_0050180), and L24e (TcSYL_0074880) and the small subunit ribosomal protein S10e (TcSYL_0114020) (Fig. 5).

### Meiosis process

**Figure 6:**
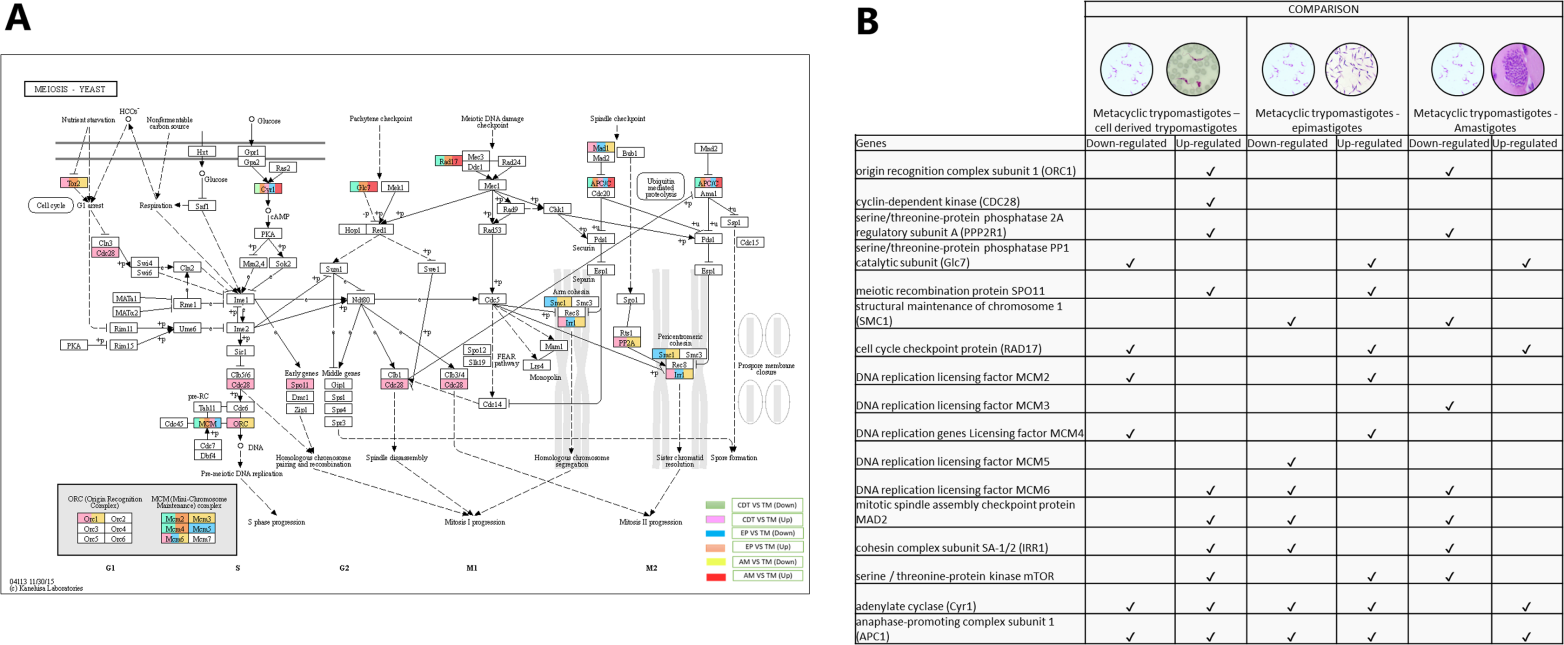
Meiosis processes. (A) Comparison of the down-regulated (green) and up-regulated (pink) pathways between metacyclic trypomastigotes and cell derived trypomastigotes. Comparison of the down-regulated (blue) and up-regulated (orange) pathways between metacyclic trypomastigotes and epimastigotes. Comparison of the down-regulated (yellow) and up-regulated (red) pathways between metacyclic trypomastigotes and amastigotes. (B) Table of down- and up-regulated genes between metacyclic trypomastigotes and amastigotes, cell derived trypomastigotes, epimastigotes. The pathway annotation pipeline using KAAS for KEGG pathway assignments was used to reconstruct the pathways and was modified to include down-regulated and up-regulated genes (Kanehisa & Goto, 2000).

Meiosis was one of the pathways in which we did not expect to find differential regulation during the *T. cruzi* life cycle. A total of 10 genes were up-regulated in MTs compared with those in CDTs, and 4 of these up-regulated genes were exclusively expressed in this stage, including origin recognition complex subunit 1 (ORC1 - TcSYL_0076530), cyclin-dependent kinase (CDC28-EC: 2.7.11.22 - TcSYL_0171170), serine/threonine-protein phosphatase 2A regulatory subunit A (PP2A - TcSYL_0110840), and the meiosis meiotic recombination protein SPO11 specific protein (K10878-TcSYL_0014080) genes. When the reads that encode to SPO11 obtained for each stage were compared, 36.2295 reads were obtained for CDTs and 60.2713 reads were obtained for MTs. Likewise, of the genes mentioned ORC1 and PP2A were down-regulated in AMs compared with MTs. No gene was found to be exclusively down-regulated between these two stages. When comparing MTs with EPs, the structural maintenance of chromosome 1 (SMC1 - TcSYL_0117100) and DNA replication licensing factor MCM5 (EC: 3.6.4.12 - TcSYL_0047860) genes were down-regulated in this process, likewise, SMC1 was down-regulated between AMs and MTs, but no exclusive up-regulated gene was observed. Serine/threonine-protein phosphatase PP1 catalytic subunit (PP1C – GLC - 7-EC: 3.1.3.16 - TcSYL_0044050), cell cycle checkpoint protein (RAD17-EC: 3.1.11.2 - TcSYL_0111040), DNA replication licensing factor MCM2 (EC: 3.6.4.12 - TcSYL_0044110), and DNA replication genes licensing factor MCM4 (EC: 3.6.4.12 - TcSYL_0155930) were down-regulated in MTs compared with those in CDTs, and they were up-regulated in MTs compared with those in EPs. Similarly, RAD17 and PP1 were down-regulated between AMs and MTs. Mitotic spindle assembly checkpoint protein MAD2 (TcSYL_0019360), cohesin complex subunit SA-1/2 (IRR1 - TcSYL_0010920), and DNA replication licensing factor MCM6 (EC: 3.6.4.12 - TcSYL_0181360) were up-regulated in MTs compared with those in CDTs, and they were down-regulated in MTs compared with those in EP and between MTs and AMs. DNA replication licensing factor MCM3 (TcSYL_0086540) was down-regulated in MTs compared with that in AMs. The serine/threonine-protein kinase mTOR (EC: 2.7.11.1-TPR2 - TcSYL_0047210) gene was up-regulated in MTs compared with that in CDTs and in MTs compared with that in EPs and down-regulated between MTs and AMs. Finally, adenylate cyclase (Cyr 1 - EC: 4.6.1.1 - TcSYL_0083140) and anaphase-promoting complex subunit 1 (APC1 - TcSYL_0139430) were down-regulated and up-regulated for all the comparisons, excepting between AMs and MTs where they only were down-regulated (Fig. 6).

### Homologous recombination

**Figure 7:**
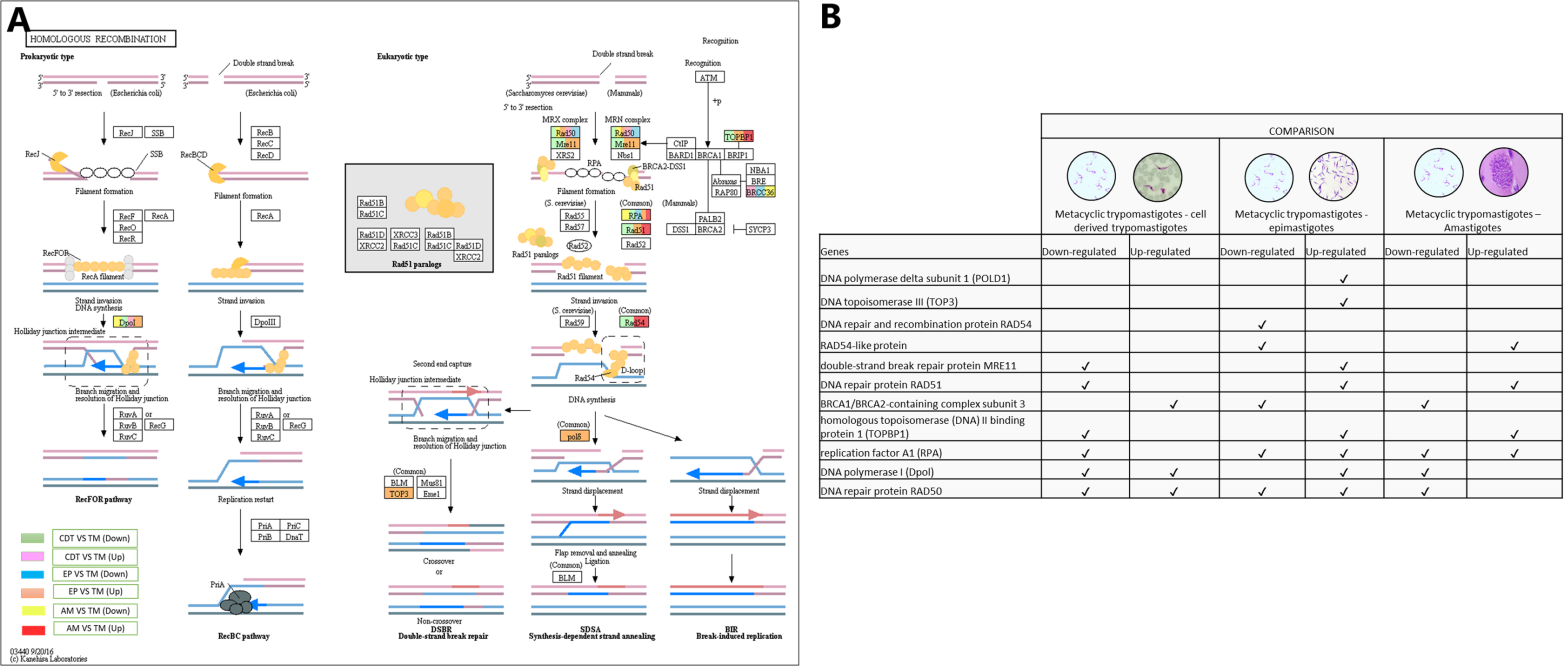
Homologous recombination processes. (A) Comparison of the down-regulated (green) and up-regulated (pink) pathways between metacyclic trypomastigotes and cell derived trypomastigotes. Comparison of the down-regulated (blue) and up-regulated (orange) pathways between metacyclic trypomastigotes and epimastigotes. Comparison of the down-regulated (yellow) and up-regulated (red) pathways between metacyclic trypomastigotes and amastigotes. (B) Table of down- and up-regulated genes between metacyclic trypomastigotes and amastigotes, cell derived trypomastigotes, epimastigotes. The pathway annotation pipeline using KAAS for KEGG pathway assignments was used to reconstruct the pathways and was modified to include down-regulated and up-regulated genes (Kanehisa & Goto, 2000).

In conjunction with the meiosis process, the signaling pathway for HR was regulated, DNA polymerase delta subunit 1 (POLD1-EC: 2.7.7.7 - TcSYL_0197030) and DNA topoisomerase III (TOP3-EC: 5.6.2.1 - TcSYL_0001820) were up-regulated in MTs compared with those in EPs, and DNA repair and recombination protein RAD54 and RAD54-like protein (EC: 3.6.4.- (TcSYL_0047980) were down-regulated in MTs compared with those in EPs and up-regulated between AMs and MT. The double-strand break (DSB) repair protein MRE11 (TcSYL_0085330), DNA repair protein RAD51 (TcSYL_0043760), and homologous topoisomerase (DNA) II binding protein 1 (TOPBP1-TcSYL_0001820) specific recombination protein were down-regulated in MTs compared with those in CDTs, and they were up-regulated in MTs compared with those in EPs and RAD51 and TOPBP1 were up-regulated between MTs and AMs. The results obtained for TOPBP1 were verified by observing that the log2 changes (fold-change) were −1.27163 and 1.71369, respectively. Replication factor A1 (RPA - TcSYL_0179190) was down-regulated in MTs compared with that in CDTs, it was down-regulated and up-regulated in MTs compared with that in EPs and down and up-regulated between MTs and AMs, whereas DNA polymerase I (Dpol-EC: 2.7.7.7 - TcSYL_0091960) was down-regulated and up-regulated in MTs compared with that in TPs, up-regulated in MTs compared with that in EP and down-regulated between MTs and AMs. Finally, DNA repair protein RAD50 (TcSYL_0043690) was found to be down-regulated and up-regulated in all the comparisons of *T. cruzi* stages, with the exception of MTs compared to AMs where it was not observed up-regulated (Fig. 7).

## Discussion

Processes related to energy metabolism (e.g., glycolysis, the pyruvate metabolism, the Krebs cycle, and oxidative phosphorylation) were differentially regulated among the four stages and were mainly down-regulated in MTs, with a greater difference observed in comparison with CDTs (Fig. 3). Metacyclogenesis is characterized by a response to strong nutritional stress stimulus, which continues in the MT stage, where energy metabolism is based on the degradation of proteins and not on glucose. However, we did not expect that the greatest difference in signaling pathways would be between MTs and CDTs, which maybe because of the manner in which CDTs acquire glucose. During intracellular infection, AMs obtain glucose from the host cell; moreover, even a decrease in the disposition process decreases the replication of amastigotes even after the differentiation into CDTs and the release of CDTs into the bloodstream. CDTs can use the substrate directly and because blood has more glucose than the rectal blister of the insect (where MTs are found), the parasite can activate energy generation from this substrate. In contrast to MTs, an increase in glucose in the infected organs and a decrease in plasma were observed, which suggests the importance of this metabolism during the AM–CDT cycle. However, we had no information about the entire curve of differentiation between these stages and at what time this metabolic differentiation occurs, and the observed results show that there is not a great difference with respect to the energetic metabolism between AMs and CDTs, later analyzes could be focused in the analysis of available reads of amastigote RNA at different times of cell culture (Contreras, Morel & Goldenberg, 1985; Hamedi et al., 2015; Kalem et al., 2018).

The signaling pathway related to RNA degradation was mainly down-regulated in MTs compared with that in CDTs, and it was up-regulated in MTs compared with that in EPs and AMs, thus demonstrating an inversely proportional relationship with the mRNA surveillance pathway. These results are of great importance considering the polycistron characteristics of *T. cruzi* where RNA regulation occurs mainly at the posttranscriptional level, and mechanisms such as RNA degradation and survival contribute greatly to its regulation mainly in stages with replicative characteristics (Fig. 3) (Clayton & Shapira, 2007; Martinez-Calvillo et al., 2010). During metacyclogenesis, there is a decrease in transcription and an increase in heterochromatin in the parasite in response to the energy deficiency it encounters. Thus, the maintenance of the proteome is generated mainly from previously transcribed RNA and is stored or free in the cytoplasm of the parasite. Conversely, it occurs in EPs where their energy metabolism is high and also presents an active replication, which requires a continuous increase of the cellular proteome and where the previously mentioned mechanisms will regulate the expression (Avila et al., 2003; Barisón et al., 2017). Therefore, we can infer that *T. cruzi* uses this tool as a source of proteome modification among the morphological stages. Another mechanism of post-transcriptional regulation is directly related to the availability and degradation of amino acids, the latter being the main form of energy in MTs (Avila et al., 2003). Although several amino acid metabolism genes were down-regulated and up-regulated, we did not identify a clear gene expression profile among the life cycle stages of *T. cruzi* (Fig. 3). However, glutathione specifically gained our attention. This molecule comprises three amino acids—glutamate, cysteine, and glycine—and was mainly down-regulated in MTs compared with that in CDTs, AMs and EPs. Glutathione is used by the parasite as trypanothione peroxidase (an antioxidant enzyme that performs detoxification) which is formed from two glutathione molecules linked to spermidine groups through the NH2 bonds of the latter and is considered a virulence factor that allows the parasite to evade the immune system (Piacenza et al., 2013). Thus, the presence of this down-regulated compound in MTs compared with that in CDTs could be related to the role of CDTs to evade the host immune system, whereas its increase in the EPs and AMs could be related to the avoidance of oxidative stress in the vector and inside the cell. This mechanism gives the parasite the ability to escape the host’s immune system or the oxidative stress present in the vector. Considering that oxidative stress mechanisms are necessary stimuli for differentiation processes such as metacyclogenesis and amastigogenesis, where EPs, AMs and CDTs are mainly exposed, would explain the higher expression of glutathione in these stages compared with that observed in MTs (Barisón et al., 2017; Nogueira et al., 2015; Tyler & Engman, 2001).

The importance of autophagy during the life cycle of *T. cruzi* and in the metacyclogenesis process has been recently suggested (Salassa & Romano, 2019; Vanrell et al., 2017). The expression of this signaling pathway was differentially regulated in all the comparisons. When reconstructing this signaling pathway and determining the DEGs, the differential expression of ATG8 was demonstrated, which is associated with one of the main autophagy process markers. It was up-regulated in MTs compared with that in EPs and up-regulated between MTs and AMs. Previous studies evaluating autophagy during metacyclogenesis demonstrated the presence of this protein during starvation, which is the main stimulus for metacyclogenesis, which would explain the decrease in its expression in amastigotes where starvation is not one of the main characteristics (Vanrell et al., 2017). Despite this, ATG3 was also up-regulated between MTs and EPs and down-regulated between MTs and AMs. ATG3 has been associated with ATG8 lipidation for autophagosome formation in encysting *Acanthamoeba*, where its presence was related to the absence of the ATG8 complex and a decrease in encysting. The presence of this up-regulated protein in MTs suggests its relationship with the completion of metacyclogenesis and the lipidation of ATG8 and the ATG8 complex formed during this process. However, because ATG8 was also up-regulated, its presence is not clear (Moon et al., 2011). On the other hand, the presence of ATG3 in *Toxoplasma gondi* is associated with the maintenance of mitochondrial homeostasis during cell division. However, MTs are not replicative, and the AMs that correspond to the replicative forms present the RNA coding for this down-regulated protein; therefore, it is possible that this protein may have different functions or specific functions in parasites or that this process is maintained as a result of nutritional stress present during metacyclogenesis and not because of cell division (Besteiro et al., 2011). ATG15 is a lipase whose function is to lyse the membrane of the autophagic body inside the lysosome for the functioning of both the compartments. This protein was up-regulated in MTs compared with that in EPs, and it was down-regulated in MTs compared with that in EPs and AMs. Although ATG15 has not been studied in depth in parasites, the reason for the differential expression profile between AMs–EPs and CDTs in comparison with MTs is not known. Therefore, we decided to evaluate the genes present in this signaling pathway with the same expression profile and found the coding gene for sec17. Sec17 is an α-SNAP protein belonging to SNARE proteins (soluble N-ethylmaleimide-sensitive factor adapting proteins receptors) related to intracellular trafficking in eukaryotes, such as trypanosomatids (Datta et al., 2018; Murungi et al., 2014). Analysis of the SNARE proteins in *T. brucei* has demonstrated their differential regulation during the life cycle of this parasite, indicating its importance in the differentiation of the parasite and thus explaining the differential expression profile observed here (Koumandou et al., 2008).

The pathway with the highest number of differentially regulated genes in all the comparisons (down-regulated and up-regulated) and stages of *T. cruzi* was related to ribosomes. However, we want to highlight the presence of related expression profiles with genes coding for specific ribosomal proteins between the parasitic stages (Fig. 5). A total of 80 ribosomal proteins have been described in eukaryotes. Similarly, the presence of ribosomal profiles in *T. cruzi* has been previously reported as a mechanism of posttranscriptional regulation in EPs and MTs. Despite this, the ribosomal profiles between AMs, CDTs and MTs have not been compared (Dalla Venezia et al., 2019; Nardelli et al., 2007; Smircich et al., 2015). Differential ribosomal profiles between the stages of *T. brucei* have been previously reported (Jensen et al., 2014; Vasquez et al., 2014). Consistent with these results, the concept of specialized ribosomes, which could determine translational activity in response to different types of stimuli related to status, development, environment, and pathologies, has recently been formulated (Dalla Venezia et al., 2019). Modifications in rRNA such as 2′-O ribose-methylation, pseudouridylation, and base methylation are associated with the function of the ribosome. However, it is not known if these rRNA modifications are related to any specific group of ribosomal proteins. Studies on these rRNAs modifications and their relationship with ribosomal proteins should be conducted to corroborate this hypothesis. The results of our study suggest the presence of a group of ribosomal proteins common to all the stages of *T. cruzi* as well as another group of proteins that are down-regulated or up-regulated between the replicative (AMs–EPs) and not replicative stages (CDTs) that could regulate the presence of proteomes specialized between the stages and confer the specific functions they exhibit (Fig. 5). However, an analysis at the proteomic level where ribosomal proteins between replicative and non-replicative stages are evaluated must be performed in order to corroborate the hypothesis proposed here.

Meiosis is one of the processes that has not been described in *T. cruzi* to date, unlike that in other trypanosomatids such as *T. brucei*, where a sexual exchange is known to occur through this form of cell reproduction (Peacock et al., 2014). Despite this, analyses at the level of single genetic markers, multilocus sequence typing (MLST), and complete genome sequencing have demonstrated the presence of genes involved in meiosis as well as events compatible with genetic exchange through a meiotic process (Berry et al., 2019; El-Sayed et al., 2005; Franzén et al., 2011; Messenger & Miles, 2015; Ramírez et al., 2012; Schwabl et al., 2019). The results obtained here demonstrated the presence of several down-regulated and up-regulated genes among the life cycle stages of *T. cruzi* related to this process. As shown in Fig. 6, only four genes were up-regulated in MTs compared with those in CDTs and SPO11 was one of the up-regulated genes. SPO11 is a specific meiosis gene, and its function is to mediate the onset of recombination and generate double chain breaks in homologous chromosomes during prophase I of meiosis I (Genois et al., 2014). To the best of our knowledge, MTs have not been associated with sexual exchange processes such as meiosis, and the only study on this process was conducted in VERO cells, where *T. cruzi* clones transfected with different antibiotics demonstrated the parasite’s ability to generate clones resistant to both antibiotics, in addition, the analysis of these clones revealed genomic characteristics generated as a result of nuclear hybridization and subsequent recombination characteristic of a meiosis process, however, the results obtained for the gene expression of AMs do not show the presence of up-regulated genes related to the process of meiosis (Gaunt et al., 2003). Therefore, to generate a reduction in ploidy through meiosis, studies on *Candida albicans* and *Giardia intestinalis* have demonstrated the ability of proteins, such as SPO11 reprogramming, to perform recombination processes during an alternative process of parasexuality, which could explain the overregulation of this protein at this stage (Carpenter et al., 2012; Forche et al., 2008; Poxleitner et al., 2008). On the other hand, “the meiosis toolkit” requires the expression of specific genes and genes that encode for proteins associated with DNA repair and other functions necessary in this process. Although we failed to detect other meiosis-specific DEGs, coding genes for proteins, such as Cdc28, Orc1, Apc1, Smc1, Rad17 and several Mcm proteins, were identified. The latter exhibited an inversely proportional gene regulation between MTs and CDTs, between MTs and AMs and between MTs and EPs. Cyclin-dependent kinase Cdc28 is necessary for meiosis initiation events, such as replication and recombination, and Orc1 is a component of the origin recognition complex. Cdc28 and Orc1 along with SPO11 participate in the formation of meiotic DNA DSBs. If we had observed the upregulation of these proteins only in MTs, we could assume that the recombination process was occurring at this stage and that it required the presence of this machinery. However, we cannot be sure that it is associated with the entire meiosis process (Fig. 6) (Akematsu et al., 2017; Benjamin et al., 2003; Farmer et al., 2012; Vader et al., 2011). Similarly, the Mcm proteins, which are a complex of seven replicative helicases, are joined via a dimer (Mcm3-5) and a trimer (Mcm4-6-7) that are connected by Mcm2. In our results, Mcm was up-regulated in MTs compared with that in CDTs, which together with SPO11, Cd28 and ORC1 indicate its participation in sexual exchange processes with MTs. It also suggests the ability of these seven proteins to differentially regulate the stages of *T. cruzi* and indicates its relationship with different processes involved in the protein conformation of the complete Mcm (Santosa et al., 2013). Future in vivo studies on cultured cells and triatomines are warranted to determine the true ability of *T. cruzi* to perform meiosis and identify the correct life stage regulating this process.

Based on the meiosis results and the regulation mainly associated with the initiation of meiosis, we decided to investigate the gene expressions among the stages of *T. cruzi* in the signaling pathway for HR. Our analysis revealed differentiation in the gene regulation of Rad proteins (Fig. 7). The following genes were identified to be up-regulated in MTs compared with those in CDTs: Rad50, MRE11 and TOPBP1, RPA, the recombinase Rad51, POLD1 and TOPO3. Rad54 was down-regulated, and with similar genetic expression, Rad50, TOPBP1, RPA, POLD1 and Rad54 when comparing the MTs with the AMs, these results demonstrate the activation of a large part of the proteins involved in the HR pathway in MTs. In addition, it is important to highlight that TOPBP1 was identified among the 50 most overregulated genes in this study, which further highlights the importance of this signaling pathway. Studies on *T. cruzi* have demonstrated a relationship between high levels of Rad51 and the presence of hybrids in the CL–Brener strain, suggesting a possible relationship between these proteins and HR in this parasite (Alves et al., 2018). Further studies on MTs evaluating the genes involved in HR are necessary to confirm this result and its possible relationship with genetic exchange processes other than meiosis, such as parasexuality. One of the limitations of this study is associated with the reads obtained from a sequencing performed in previous years. Subsequent studies must focus on the follow-up of a strain or clone of *T. cruzi* throughout its life cycle in the same period of time to reduce possible external variables.

## Conclusions

From the results obtained here, we can conclude that there is a difference in gene expression among the stages of *T. cruzi*. Considering the regulation of energy metabolism from glucose, the maintenance and survival of mRNA, the participation of the autophagy processes, not only as stimuli in the metacyclogenesis but also as processes present throughout the parasite’s life cycle, and the ribosomal profiles among the stages, we can infer its importance in the posttranscriptional regulation and proteome found in the different stages of *T. cruzi*. We can also infer a genetic exchange process in the MTs where SPO11 and Rad51 play a fundamental role in the development of DSBs and HR. To the best of our knowledge, this is the first study where RNA sequencing was used as a tool to analyze the expression profiles present in MTs and their respective comparison with all stages of *T. cruzi* cycle. The results obtained here open a window of knowledge toward processes and pathways of signaling regulated in the infectious stage of the parasite and the life cycle of *T. cruzi* in general. The response to different microenvironments and types of stress during their life cycle provides an opportunity to control the transmission of this parasite and improve our understanding of the drivers of *T. cruzi* cell biology.

## Supplemental Information

10.7717/peerj.8947/supp-1 Supplemental Information 1 Comparison between biological replicates.

10.7717/peerj.8947/supp-2 Supplemental Information 2 Reads details. Information on the access number of RNA reads for epimastigotes (EPs), amastigotes (AMs), cell-derived trypomastigotes (CDTs) obtained from the ENA platform for comparison and metacyclic trypomastigotes (MTs). (xlsx)

10.7717/peerj.8947/supp-3 Supplemental Information 3 DEG statistics. Down-regulated and up-regulated DEGs I metacyclic trypomastigotes (MTs) compared to epimastigotes (EPs) and metacyclic trypomastigotes (MTs) compared to procyclic trypomastigotes (PTs) using the cuffdiff tool in Cufflinks. (xlsx)

10.7717/peerj.8947/supp-4 Supplemental Information 4 Gene ontology. Down-regulated and up-regulated gene ontology from DEGs metacyclic trypomastigotes (MTs) compared to epimastigotes (EPs), metacyclic trypomastigotes (MTs) compared to procyclic trypomastigotes (PTs) and metacyclic trypomastigotes (MTs) compared to amastigotes (AMs) using the eupath tool. (xlsx)

## Additional Information and Declarations

### Data Availability

The following information was supplied regarding data availability: Data is available at the European Nucleotide Archive (ENA): PRJNA251583 (SAMEA5925497) and PRJEB33521 (SAMEA5925496, SAMEA5925498).
