# Supplementary figures and images for: Transcriptomic changes across the life cycle of Trypanosoma cruzi II
Source: PeerJ. 2020 May 14;8:e8947. doi: 10.7717/peerj.8947 (PMC7231504; doi:10.7717/peerj.8947)

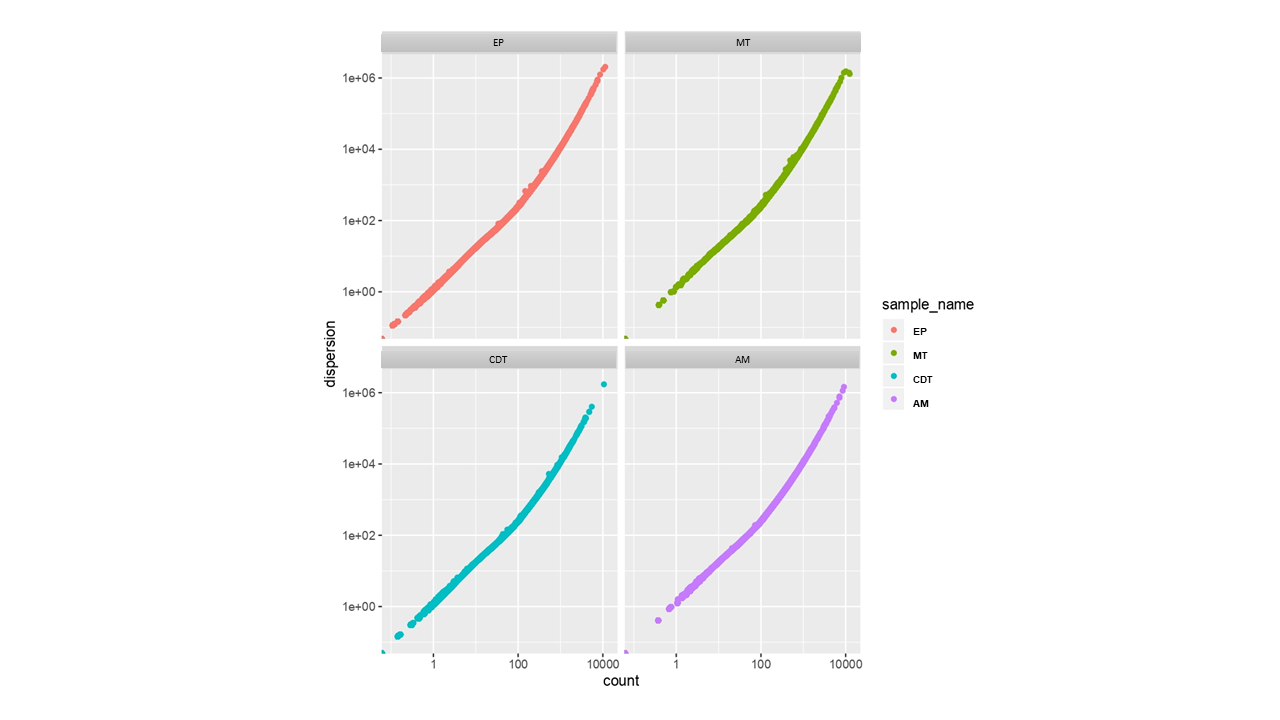

Supplement: Supplemental Information 1 [file peerj-08-8947-s001.tif]
